# SNP-SNP interactions of oncogenic long non-coding RNAs HOTAIR and HOTTIP on gastric cancer susceptibility

**DOI:** 10.1038/s41598-020-73682-0

**Published:** 2020-10-07

**Authors:** Esmat Abdi, Saeid Latifi-Navid, Saber Zahri, Vahid Kholghi-Oskooei, Behdad Mostafaiy, Abbas Yazdanbod, Farhad Pourfarzi

**Affiliations:** 1 Department of Biology, Faculty of Sciences, University of Mohaghegh Ardabili, 5619911367 Ardabil, Iran; 2 Department of Laboratory Sciences, School of Paramedical Sciences, Torbat Heydariyeh University of Medical Sciences, 9516915169 Torbat Heydariyeh, Iran; 3 Health Sciences Research Center, Torbat Heydariyeh University of Medical Sciences, 9516915169 Torbat Heydariyeh, Iran; 4 Department of Statistics, Faculty of Sciences, University of Mohaghegh Ardabili, 5619911367 Ardabil, Iran; 5 Digestive Disease Research Center, Ardabil University of Medical Sciences, 5618953141 Ardabil, Iran

**Keywords:** Cancer, Gastroenterology, Oncology, Risk factors

## Abstract

Genetic variants within oncogenic long non-coding RNAs HOTAIR and HOTTIP may affect their gene expression levels, thereby modifying genetic susceptibility to gastric cancer (GC). In a hospital-based study in Ardabil—a very high-risk area in North-West Iran, 600 blood samples from 300 GC patients and 300 healthy controls were recruited for genotyping. Seven HOTAIR (i.e., rs17720428, rs7958904, rs1899663, and rs4759314) and HOTTIP (i.e., rs3807598, rs17501292, and rs1859168) ‘tag’ single nucleotide polymorphisms (SNPs) were genotyped by the Infinium HTS platform. The rs17720428, rs7958904, and rs1899663 tagSNPs significantly increased GC risk under dominant models by 1.5-, 1.57-, and 1.5-fold, respectively. The G-C-T-A haplotype of HOTAIR tagSNPs increased the risk of GC by 1.31-fold. No significant association was found between HOTTIP SNPs and the risk of GC. HOTAIR and HOTTIP variants were also not associated with any clinicopathologic characteristics. The SNP-SNP interaction of HOTAIR rs17720428/rs7958904 with HOTTIP rs1859168 was associated with an increased risk of GC (rs17720428 TG-rs1859168 CC, OR = 1.76; rs7958904 GC-rs1859168 CC, OR = 1.85; rs7958904 CC-rs1859168 CC, OR = 1.86). Interestingly, the SNP-SNP interaction of HOTAIR rs1899663 with HOTTIP rs1859168 strongly increased the risk of GC (rs1899663 GT-rs1859168 CC, OR = 4.3; rs1899663 TT-rs1859168 CC, OR = 9.37; rs1899663 TT-rs1859168 CA, OR = 6.59). We showed that the HOTAIR rs17720428, rs7958904, and rs1899663 tagSNPs and their interactions with the HOTTIP rs1859168 polymorphism significantly increased the risk of GC. Specifically, novel SNP-SNP interactions between HOTAIR and HOTTIP tagSNPs have a larger impact than individual SNP effects on GC risk, thereby providing us with valuable information to reveal potential biological mechanisms for developing GC.

## Introduction

Gastric cancer (GC) is a prevalent disease of the digestive system^1–3^. It is the fifth prevalent kind of cancer (5.7%) and the third cause of cancer-related mortality (8.2%)^4^. In spite of the incidence decline in some parts of the world, GC is a crucial challenge since most incidences are diagnosed at advanced stages, following poor prognosis^5,6^. Thus, reliable biomarkers of GC must be identified for effective therapy, early diagnosis, and prognosis evaluation. Single nucleotide polymorphisms (SNPs) have profound to have influences on gene function and expression, and contribute to carcinogenesis. Studies of genome-wide association which scan the whole genome for prevalent genetic variants have shown over 450 SNPs related to susceptibility to different cancer types^7^. Only 7% of these loci are in protein-coding areas, but 93% are located in non-coding areas^8,9^. Non-coding RNAs are the major regulators of some biological processes, including translation, transcription, epigenetic gene expression, splicing, cell cycle, embryogenesis, stem cell pluripotency and reprogramming, and the immune response regulation^10,11^. Aberrant expression of long non-coding RNAs (lncRNAs) may bring about different cancers^12–14^. Various lncRNAs are associated with different cancer types^15–19^. HOX transcript antisense RNA (HOTAIR)—a well-known oncogenic lncRNA—is highly expressed in GC tissues and has been recognized as a critical prognostic biomarker for major cancers, including GC. HOTAIR inhibition not only reduces tumor invasiveness but also reverses EMT in GC cells by regulating N-cadherin, E-cadherin, vimentin, and a transcription factor snail. HOTAIR targets miR-126 to activate the multidrug resistance-associated protein 1/phosphatidylinositol 3-kinase (PI3-K)/Akt and thus promotes cisplatin resistance in GC. Specifically, it directly inhibits miR126, promoting the expression of PI3-K regulatory subunit beta and vascular endothelial growth factor A. Therefore, HOTAIR-targeted therapies may potentially improve prognosis and survival of patients suffering from GC^15,20–22^. The HOXA transcript at the distal tip (HOTTIP), transcribed from the 5′ tip of HOXA cluster, is a cancer-related lncRNA^23,24^. Recruiting HOXA13–HOTAIR and HOXA13–HOTTIP to different sites in the promoter of bone morphogenetic protein 7 (BMP7) is critical for the oncogenic fate of the human gastric cells^24^. HOTTIP was overexpressed significantly in cell lines of GC; HOTTIP down-regulation would hinder cell proliferation, degrade cell invasion and migration, and develop cell apoptosis^25^. Ardabil Province is a very high-risk area in North-West Iran (ASRs, 51.8/100,000 and 24.9/100,000 for males and females, respectively), with one of the highest cardia GC (CGC) rates worldwide. Hence, in a case–control study from Ardabil, we genotyped seven HOTAIR (i.e., rs17720428, rs7958904, rs1899663, and rs4759314) and HOTTIP (i.e., rs3807598, rs17501292, and rs185916) tagSNPs to assess their associations with the risk of GC. In addition, to perform data mining regarding the SNP-SNP interactions, all possible pair combinations between all of the HOTAIR and HOTTIP SNPs in relation to GC susceptibility were analyzed.

## Results

### General characteristics of the study subjects

Each of GC and control groups consisted of 300 subjects, of whom 74.7% were males. The average age (mean ± SD (min–max)) was 66.54 ± 10.43 (34–88) and 66.48 ± 9.71 (38–91) years for cases and controls, respectively. Age and gender were not significantly different between the case and control groups (*p* = 0.37 and *p* = 1.00, respectively), which indicated that these groups were matched well with respect to these parameters. The genotype distributions of SNPs in cases and controls met the Hardy–Weinberg equilibrium conditions (Table 1(. The prevalence rate of GC patients based on the anatomic site of the tumor origin was 52.5% with CGC, 35% with NCGC, and 12.5% with both CGC and NCGC. According to histopathologic features, the prevalence rate of the intestinal-, diffuse-, and indeterminate-type GC was 49.7%, 19.7%, and 30.6%, respectively. Moreover, the 0.8, 5.2, 45.5 and 48.5% of patients were diagnosed at stages I, II, III, and IV, respectively. The clinical and demographic characteristics of the subjects are presented in Table 2.

**Table 1:** Exact test for Hardy–Weinberg equilibrium (*p-value*).

	rs17720428	rs7958904	rs1899663	rs4759314	rs3807598	rs17501292	rs1859168
All subjects	0.37	0.87	0.73	1	0.12	0.06	0.43
Controls	0.61	0.4	0.61	1	0.56	0.06	0.36
Patients	0.23	0.56	0.23	1	0.11	0.07	0.51

**Table 2:** Baseline characteristics of total 300 GC patients and 300 cancer-free controls.

**Case**		
Age (mean ± SD (min–max))	**–**	66.54 ± 10.43 (34–88)
Gender	Male	74.7%
	Female	25.3%
Tumor origin	Cardia	52.5%
	Cardia and non-cardia	12.5%
	Non-cardia	35%
Pathology	Intestinal-type GC	49.7%
	Diffuse-type GC	19.7%
	Other	30.6%
TNM Stage	I	0.8%
	II	5.2%
	III	45.5%
	IV	48.5%
T	T1	9.4%
	T2	18.1%
	T3	24.2%
	T4	48.3%
N	N0	1.1%
	N1	2.6%
	N2	35.3%
	N3	60.9%
M	M0	51.9%
	M1	48.1%
**Control**		
Age (mean ± SD (min–max))	**–**	66.48 ± 9.71 (38–91)
Gender	Male	74.7
	Female	25.3

### The association between HOTAIR/HOTTIP tagSNPs and GC risk

The average call rate for the 600 analyzed samples was 99.84%, showing high call rates and high reproducibility. Three SNPs of HOTAIR (i.e., rs17720428; rs7958904; rs1899663) were associated with an increased risk of GC. It was found that the rs17720428 polymorphism was associated with the risk of GC, assuming allelic, dominant, and log-additive models of inheritance. The findings revealed that the rs17720428 G allele was significantly associated with the increased risk of GC (G vs. T, OR = 1.27, 95% CI = 1.01–1.61; *p* = 0.04). In the dominant model, subjects carrying the TG + GG genotype of rs17720428, as compared with those carrying the TT genotype, had a significantly higher risk of GC (OR = 1.5, 95% CI: 1.08–2.1; *p* = 0.01).

The rs7958904 SNP was associated with the risk of GC in allelic, co-dominant, dominant, and log-additive models of inheritance. The rs7958904 C allele was significantly associated with the increased risk of GC (C vs. G, OR = 1.31, 95% CI: 1.04–1.65; *p* = 0.02). Subjects carrying the CC or GC genotype of rs7958904, as compared with those carrying the GG genotype in the co-dominant model, showed an increased risk of GC (CC vs. GG, OR = 1.54, 95% CI: 1.07–2.22 and GC vs. GG, OR = 1.64, 95% CI: 1.03–2.62; *p* = 0.04). In the dominant model, subjects carrying the GC + CC genotype of rs7958904 showed an increased risk of GC in comparison with those carrying the GG genotype (OR = 1.57, 95% CI: 1.1–2.22; *p* = 0.01).

The rs1899663 SNP was associated with the risk of GC, assuming allelic, dominant and log-additive models of inheritance. The findings indicated that the rs1899663 T allele was significantly associated with the increased risk of GC (T vs. G, OR = 1.27, 95% CI: 1.01–1.61; *p* = 0.04). The GT + TT genotype of rs1899663, in comparison with GG genotype, had a significantly higher risk of GC in the dominant model (OR = 1.5, 95% CI: 1.08–2.08; *p* = 0.02). No significant associations were observed between the rs4759314 SNP and GC susceptibility (Table 3). No evidence regarding the association between the HOTTIP tagSNPs (i.e., rs3807598, rs17501292, and rs1859168) and GC risk was found in any of the genetic models (*p* > 0.05; Table 4). The HOTAIR and HOTTIP variants were not associated with any clinicopathologic characteristics (Table 5). Moreover, the frequency of each HOTAIR/HOTTIP tagSNP did not show a significant difference between patients having stage I-II and stage III-IV disease (Table 6).

**Table 3:** Genotype and allele frequencies of HOTAIR SNPs in cases and controls, and genotype- and allelotype-specific risks.

Locus	Model	Genotype	Patients	Controls	Odds Ratio	*p*-value
**rs17720428**						
	Allele	T	359 (59.8%)	393 (65.5%)	1	0.04
		G	241 (40.21%)	207 (34.5%)	1.27 (1.01–1.61)	
	Codominant	TT	102 (34%)	131 (43.7%)	1	0.05
		TG	155 (51.7%)	131 (43.7%)	1.52 (1.07–2.15)	
		GG	43 (14.3%)	38 (12.6%)	1.45 (0.87–2.41)	
	Dominant	TT	102 (34%)	131 (43.7%)	1	0.01
		TG + GG	198 (66%)	169 (56.3%)	1. 5 (1.08–2.1)	
	Recessive	TT + TG	257 (85.7%)	262 (87.4%)	1	0.55
		GG	43 (14.3%)	38 (12.6%)	1.15 (0.72–1.84)	
	Overdominant	TT + GG	145 (48.3%)	169 (56.3%)	1	0.05
		TG	155 (51.7%)	131 (43.7%)	1.38 (1–1.9)	
	Log-Additive				1.28 (1.01–1.63)	0.04
**rs7958904**						
	Allele	G	315 (52.7%)	355 (59.4%)	1	0.02
		C	283 (47.3%)	243 (40.6%)	1.31 (1.04–1.65)	
	Codominant	GG	80 (26.8%)	109 (36.5%)	1	0.04
		GC	155 (51.8%)	137 (45.8%)	1.54 (1.07–2.22)	
		CC	64 (21.4%)	53 (17.7%)	1.64 (1.03–2.62)	
	Dominant	GG	80 (26.8%)	109 (36.5%)	1	0.01
		GC + CC	219 (73.2%)	190 (63.5%)	1.57 (1.1–2.22)	
	Recessive	GG + GC	235 (78.6%)	246 (82.3%)	1	0.26
		CC	64 (21.4%)	53 (17.7%)	1.26 (0.84–1.9)	
	Overdominant	GG + CC	144 (48.2%)	162 (54.2%)	1	0.14
		GC	155 (51.8%)	137 (45.8%)	1.27 (0.92–1.75)	
	Log-Additive				1.31 (1.04–1.65)	0.02
**rs1899663**						
	Allele	G	358 (59.9%)	393 (65.5%)	1	0.04
		T	240 (40.1%)	207 (34.5%)	1.27 (1.01–1.61)	
	Codominant	GG	102 (34.1%)	131 (43.7%)	1	0.06
		GT	154 (51.5%)	131 (43.7%)	1.51 (1.06–2.13)	
		TT	43 (14.4%)	38 (12.7%)	1.45 (0.87–2.41)	
	Dominant	GG	102 (34.1%)	131 (43.7%)	1	0.02
		GT + TT	197 (65.9%)	169 (56.3%)	1.5 (1.08–2.08)	
	Recessive	GG + GT	256 (85.6%)	262 (87.3%)	1	0.54
		TT	43 (14.4%)	38 (12.7%)	1.16 (0.72–1.85)	
	Overdominant	GG + TT	145 (48.5%)	169 (56.3%)	1	0.05
		GT	154 (51.5%)	131 (43.7%)	1.37 (0.99–1.89)	
	Log-Additive				1.28 (1.01–1.62)	0.04
**rs4759314**						
	Allele	A	586 (97.7%)	590 (98.3%)	1	0.41
		G	14 (2.3%)	10 (1.7%)	1.41 (0.62–3.2)	
	Codominant	AA	286 (95.3%)	290 (96.7%)	1	0.4
		AG	14 (4.7%)	10 (3.3%)	1.42 (0.62–3.25)	
		GG	–	–	–	
	Dominant	–	–	–	–	
		–	–	–	–	
	Recessive	–	–	–	–	
		–	–	–	–	
	Overdominant	–	–	–	–	
		–	–	–	–	

OR, odds ratio; CI, confidence interval; SNPs, single nucleotide polymorphisms.

**Table 4:** Genotype and allele frequencies of HOTTIP SNPs in cases and controls, and genotype- and allelotype-specific risks.

Locus	Model	Genotype	Patients	Controls	Odds Ratio	*p*-value
**rs3807598**						
	Allele	C	315 (52.7%)	308 (51.5%)	1	0.68
		G	283 (47.3%)	290 (48.5%)	0.95 (0.76–1.2)	
	Codominant	CC	90 (30.1%)	82 (27.4%)	1	0.71
		CG	135 (45.2%)	144 (48.2%)	0.85 (0.58–1.24)	
		GG	74 (24.7%)	73 (24.4%)	0.92 (0.59–1.35)	
	Dominant	CC	90 (30.1%)	82 (27.4%)	1	0.47
		CG + GG	209 (69.9%)	217 (72.6%)	0.89 (0.61–1.25)	
	Recessive	CC + CG	225 (75.3%)	226 (75.6%)	1	0.92
		GG	74 (24.7%)	73 (24.4%)	1.02 (0.7–1.48)	
	Overdominant	CC + GG	164 (54.8%)	155 (51.8%)	1	0.46
		CG	135 (45.2%)	144 (48.2%)	0.89 (0.64–1.22)	
	Log-Additive				0.96 (0.77–1.19)	0.69
**rs17501292**						
	Allele	T	492 (0.83)	514 (0.86)	1	0.1
		G	104 (0.17)	84 (0.14)	1.29 (0.95–1.77)	
	Codominant	TT	208 (69.8%)	222 (74.3%)	1	0.29
		TG	76 (25.5%)	70 (23.4%)	1.16 (0.8–1.69)	
		GG	14 (4.7%)	7 (2.3%)	2.13 (0.84–5.39)	
	Dominant	TT	208 (69.8%)	222 (74.3%)	1	0.23
		TG + GG	90 (30.2%)	77 (25.7)	1.24 (0.87–1.78)	
	Recessive	TT + TG	284 (95.3%)	292 (97.7%)	1	0.12
		GG	14 (4.7%)	7 (2.3%)	2.06 (0.82–5.17)	
	Overdominant	TT + GG	222 (74.5%)	229 (76.6%)	1	0.55
		TG	76 (25.5%)	70 (23.4%)	1.12 (0.77–1.63)	
	Log-Additive				1.27 (0.94–1.72)	0.12
**rs1859168**						
	Allele	C	569 (95.2%)	561 (93.5%)	1	0.22
		A	29 (4.8%)	39 (6.5%)	0.73 (0.45–1.2)	
	Codominant	CC	271 (90.7%)	263 (87.7%)	1	0.48
		CA	27 (9%)	35 (11.7%)	0.75 (0.44–1.27)	
		AA	1 (0.3%)	2 (0.6%)	0.48 (0.04–5.38)	
	Dominant	CC	271 (90.7%)	263 (87.7%)	1	0.24
		CA + AA	28 (9.3%)	37 (12.3%)	0.73 (0.48–1.23)	
	Recessive	CC + CA	298 (90.7%)	298 (99.4%)	1	0.56
		AA	1 (0.3%)	2 (0.6%)	0.5 (0.04–5.54)	
	Overdominant	CC + AA	272 (91%)	265 (88.3%)	1	0.29
		CA	27 (9%)	35 (11.7%)	0.75 (0.44–1.28)	
	Log-additive				0.74 (0.45–1.21)	0.22

OR, odds ratio; CI, confidence interval; SNPs, single nucleotide polymorphisms.

**Table 5:** Subgroup analysis of clinical characteristics for the association of SNPs with GC risk.

	Gender			Tumor origin				Pathology			
	Male	Female	*p*-value	Cardia	Cardia/Non-cardia	Non-cardia	*p*-value	Intestinal-type GC	Diffuse-type GC	Others	***p-value***
**rs17720428**			0.19				0.58				0.69
TT	80 (78.4%)	22 (21.6%)		56 (33.3%)	3 (66.7%)	43 (42.2%)		48 (47.1%)	20 (19.6%)	34 (33.3%)	
TG	109 (70.3%)	46 (29.7%)		78 (50.6%)	3 (1.9%)	73 (47.4%)		81 (52.3%)	32 (20.6%)	42 (27.1%)	
GG	35 (81.4%)	8 (18.6%)		27 (62.8%)	1 (2.3%)	15 (39.9%)		20 (46.5%)	7 (16.3%)	16 (37.2%)	
**rs7958904**			0.31				0.54				0.85
GG	62 (77.5%)	18 (22.5%)		45 (56.3%)	2 (2.5%)	33 (41.3%)		37 (46.2%)	16 (20%)	27 (33.8%)	
GC	110 (71%)	45 (29%)		80 (51.9%)	2 (1.3%)	72 (46.8%)		80 (51.6%)	32 (20.7%)	43 (27.7%)	
CC	51 (79.7%)	13 (20.3%)		35 (54.7%)	3 (4.7%)	26 (40.6%)		32 (50%)	11 (17.2%)	21 (32.8%)	
**rs1899663**			0.18				0.63				0.64
GG	80 (78.4%)	22 (21.6%)		55 (53.9%)	3 (2.9%)	44 (43.1%)		48 (47.1%)	20 (19.6%)	34 (33.3%)	
GT	108 (70.1%)	46 (29.9%)		78 (51%)	3 (2%)	72 (47.1%)		81 (52.6%)	32 (20.8%)	42 (26.6%)	
TT	35 (81.4%)	8 (18.6%)		27 (62.8%)	1 (2.3%)	15 (34.9%)		20 (46.5%)	7 (16.3%)	16 (37.2%)	
**rs4759314**			0. 33				0.049				0.82
AA	212 (74.1%)	74 (25.9%)		155 (54.4%)	5 (1.8%)	125 (43.9%)		141 (49.3%)	57 (19.9%)	88 (30.8%)	
AG	12 (85.7%)	2 (14.3%)		6 (42.9%)	2 (14.3%)	6 (42.9%)		8 (57.1%)	2 (14.3%)	4 (28.6%)	
**rs3807598**			0.049				0.29				0.2
CC	61 (67.8%)	29 (32.2%)		43 (48.3%)	2 (2.3%)	44 (49.4%)		50 (55.6%)	19 (21.1%)	21 (23.3%)	
CG	110 (81.5%)	25 (18.5%)		82 (60.7%)	3 (22.3%)	50 (37%)		58 (43%)	27 (20%)	50 (37%)	
GG	52 (70.3%)	22 (29.7%)		36 (48.6%)	2 (2.7%)	36 (48.6%)		40 (54.1%)	13 (17.6%)	21 (28.4%)	
**rs17501292**			0.08				0.96				0.44
TT	158 (76%)	50 (24%)		110 (52.9%)	5 (2.4%)	99 (44.7%)		97 (46.6%)	44 (21.2%)	67 (32.2%)	
TG	59 (77.6%)	17 (22.4%)		42 (56%)	2 (2.7%)	31 (41.3%)		43 (56.6%)	14 (18.4%)	19 (25%)	
GG	7 (50%)	7 (50%)		7 (50%)	0 (0%)	7 (50%)		8 (57.1%)	1 (7.1%)	5 (35.7%)	
**rs1859168**			0.28				0.74				0.63
CC	206 (76%)	65 (24%)		145 (53.7%)	6 (2.2%)	119 (44.1%)		134 (49.4%)	53 (19.6%)	84 (31%)	
CA	17 (63%)	10 (37%)		14 (51.9%)	1 (3.7%)	12 (44.4%)		14 (51.9%)	6 (22.2%)	7 (25.9%)	
AA	1 (100%)	0 (0%)		1 (100%)	0 (0%)	0 (0%)		0 (0%)	0 (0%)	1 (100%)

**Table 6:** Relationship of clinical stage with HOTAIR/HOTTIP polymorphisms in GC patients.

	Stage		
	I, II	III, IV	*p-*value
**rs17720428**			0.23
TT	7 (7.6%)	85 (92.4%)	
TG	5 (3.7%)	130 (96.3%)	
GG	4 (10.3%)	35 (89.7%)	
**rs7958904**			0.08
GG	6 (8.5%)	65 (91.5%)	
GC	4 (2.9%)	132 (97.1%)	
CC	6 (10.3%)	52 (89.7%)	
**rs1899663**			0.23
GG	7 (7.6%)	85 (92.4%)	
GT	5 (3.7%)	130 (96.3%)	
TT	4 (10.3%)	35 (89.7%)	
**rs4759314**			0.85
AA	15 (6%)	237 (94%)	
AG	1 (7.1%)	13 (92.9%)	
**rs3807598**			0.98
CC	5 (6.2%)	75 (93.8%)	
CG	7 (5.7%)	115 (94.3%)	
GG	4 (6.3%)	59 (93.7%)	
**rs17501292**			0.44
TT	9 (4.9%)	176 (95.1%)	
TG	6 (9.2%)	59 (90.8%)	
GG	1 (7.1%)	13 (92.9%)	
**rs1859168**			0.41
CC	16 (6.7%)	224 (93.3%)	
CA	0 (0%)	24 (100%)	
AA	0 (0%)	1 (100%)	

### The association of haplotype in two lncRNA genes with GC risk

According to Table 7, the results of haplotype analysis showed that the G-C-T-A haplotype of HOTAIR rs17720428, rs7958904, rs1899663, and rs4759314, respectively, increased the risk of GC by 1.31-fold (95% CI: 1.03–1.67; *p* = 0.029). No haplotype of the three HOTTIP tagSNPs was associated with the risk of GC (*p* > 0.05).

**Table 7:** Association of the haplotype of HOTAIR/HOTTIP gene with GC risk were calculated using the SNPStats.

*HOTAIR*	rs17720428	rs7958904	rs1899663	rs4759314	Frequency in control group	Frequency in patients group	OR (95%CI)	*p*-value
	T	G	G	A	0.5948	0.5281	1	-
	G	C	T	A	0.3432	0.4	1.31 (1.03—1.67)	0.029
	T	C	G	A	0.0453	0.0469	1.16 (0.67—2.01)	0.61
	T	C	G	G	0.0149	0.0233	1.72 (0.73—4.08)	0.22
	G	C	T	G	0.0018	–	–	–
	G	C	G	A	-	0.0017	–	–
***HOTTIP***	**rs3807598**	**rs17501292**	**rs1859168**	–				
	G	T	C	–	0.4843	0.4696	1	-
	C	T	C	–	0.3181	0.3121	1.01 (0.78—1.31)	0.93
	C	G	C	–	0.1326	0.1676	1.28 (0.92—1.77)	0.14
	C	T	A	–	0.0571	0.0444	0.77 (0.45—1.32)	0.34
	C	G	A	–	0.0079	0.004	0.90 (0.16—4.94)	0.9
	G	G	C	–	–	0.0024	–	–

https://www.snpstats.net/start.htm based on the expectation maximization algorithm.

### SNP-SNP interaction models for lncRNA polymorphisms

To perform data mining regarding the SNP-SNP interactions, all possible pair combinations between all of the HOTAIR and HOTTIP tagSNPs were analyzed. The interaction of HOTAIR rs17720428 TG with HOTTIP rs1859168 CC potentially increased the risk of GC (OR = 1.76, 95% CI: 1.22–2.54; *p* = 0.003). In addition, the interaction of HOTAIR rs7958904 with HOTTIP rs1859168 potentially increased the risk of GC (rs7958904 GC-rs1859168 CC, OR = 1.85, 95% CI: 1.25–2.73, *p* = 0.002; rs7958904 CC-rs1859168 CC, OR = 1.86, 95% CI: 1.14–3.06, *p* = 0.01). Interestingly, the interaction of HOTAIR rs1899663 with HOTTIP rs1859168 strongly increased the risk of GC (rs1899663 GT-rs1859168 CC, OR = 4.3, 95% CI: 2.75–6.7; rs1899663 TT-rs1859168 CC, OR = 9.37, 95% CI: 5.43–16.18; rs1899663 TT-rs1859168 CA, OR = 6.59, 95% CI: 2.12–20.51; all the *p*-values were < 0.001.) (Table 8).

**Table 8:** The two-way interaction of HOTAIR and HOTTIP polymorphism in the risk of GC.

SNP-SNP interaction	SNP Genotype		Patients	Controls	Odds Ratio	*p*-value
**rs17720428 and rs1859168**	***HOTAIR***	***HOTTIP***				
	TT	CC	91	122	1	
	TT	CA	10	9	1.49 (0.58–3.81)	0.4
	TT	AA	1	0		
	TG	CC	143	109	1.76 (1.22–2.54)	0.003
	TG	CA	12	21	0.77 (0.39–1.64)	0.49
	TG	AA	0	1		
	GG	CC	37	32	1.55 (0.9–2.67)	0.11
	GG	CA	5	5	1.34 (0.38–4.77)	0.65
	GG	AA	0	1		
**rs7958904 and rs1859168**	***HOTAIR***	***HOTTIP***				
	GG	CC	70	103	1	
	GG	CA	9	6	2.2 (0.75–6.47)	0.41
	GG	AA	1	0		
	GC	CC	143	114	1.85 (1.25–2.73)	0.002
	GC	CA	12	22	0.8 (0.37–1.73)	0.57
	GC	AA	0	1		
	CC	CC	57	45	1.86 (1.14–3.06)	0.01
	CC	CA	6	7	1.26 (0.4–3.91)	0.69
	CC	AA	0	1		
**rs1899663 and rs1859168**	***HOTAIR***	***HOTTIP***				
	GG	CC	37	122	1	
	GG	CA	5	9	1.83 (0.59–5.8)	0.3
	GG	AA				
	GT	CC	142	109	4.3 (2.75–6.7)	< 0.001
	GT	CA	12	21	1.88 (0.85–4.19)	0.12
	GT	AA	0	1		
	TT	CC	91	32	9.37 (5.43–16.18)	< 0.001
	TT	CA	10	5	6.59 (2.12–20.51)	< 0.001
	TT	AA	1	1	3.3 (0.2–54.01)	0.38

### The potential impact of each SNP on the establishment or destruction of the miRNA binding site

Bioinformatic analysis showed that the HOTAIR rs17720428/rs7958904 and HOTTIP rs17501292 tagSNPs cause miRNA target gain and loss. Moreover, the HOTTIP rs1859168 polymorphism could lead to miRNA target gain while the rs3807598 polymorphism could lead to miRNA target loss. For the HOTAIR rs1899663 and rs4759314 tagSNPs, no miRNA target gain or loss was recognized (Table 9).

**Table 9:** The potential impact of each SNP on the establishment or destruction of the miRNA binding site.

	SNP causes miRNA target gain	SNP causes miRNA target loss
rs17720428	hsa-miR-6513-5p, hsa-miR-450b-3p, hsa-miR-5089-5p, hsa-miR-769-3p,	hsa-miR-5004-3p,
rs7958904	hsa-miR-6721-5p, hsa-miR-1203	hsa-miR-4750-3p, hsa-miR-615-3p, hsa-miR-6742-5p
rs1899663	–	–
rs4759314	–	–
rs3807598	–	hsa-miR-3115
rs17501292	hsa-miR-8080	hsa-miR-1252-5p, hsa-miR-651-3p, hsa-miR-5681a
rs1859168	hsa-miR-5699-5p, hsa-miR-874-5p, hsa-miR-506-5p, hsa-miR-216a-5p	–

## Discussion

Evidences have demonstrated that the aberrant expression of lncRNAs may develop various malignancies^26,27^. Moreover, polymorphisms in lncRNAs may influence their expression and bring about GC susceptibility^28,29^. SNPs in lncRNAs may affect different biological processes through affecting biological pathways. Studies have confirmed the roles of lncRNAs as critical regulators of tumorigenesis^30^. The current study explored whether the tagSNPs of HOTAIR (i.e., rs17720428, rs7958904, rs1899663, and rs4759314) and HOTTIP (i.e., rs3807598, rs17501292, and rs185916) affect GC development. The G allele and TG + GG genotype of rs17720428 in HOTAIR significantly increased the risk of GC (G vs. T, OR = 1.27; TG + GG vs. TT, OR = 1.5, respectively). We also showed that the T allele and GT + TT genotype of rs1899663 in HOTAIR were correlated with the higher GC risk (T vs. G, OR = 1.27; GT + TT vs. GG, OR = 1.5).

The C allele of rs7958904 in HOTAIR was correlated with the increased risk of GC (C vs. G, OR = 1.31). Patients carrying the GC or CC genotype of rs7958904 had considerably increased the risk of GC compared to those carrying the GG genotype (OR = 1.54 and GC vs. GG, OR = 1.64, respectively). In addition, subjects carrying the GC + CC genotype of rs7958904 possessed a meaningful increased risk of GC compared to individuals carrying the GG genotype (OR = 1.57). It has been shown that the HOTAIR rs7958904 CC genotype associates with the higher cervical cancer risk in comparison to the GG/GC genotypes (OR = 1.57). TCGA database revealed that the cervical cancer tissues with the rs7958904 CC genotype had increased the expression of HOTAIR compared to those with GG genotype. Hence, HOTAIR rs7958904 may affect cervical cancer susceptibility by the modulation of CC cell proliferation^31^. It is the possibility of additive roles of genetic and environmental factors with SNPs and understanding gene–gene/gene-environmental interactions are prerequisites for highly effective prevention.

Du et al. demonstrated that the HOTAIR SNP rs4759314 was significantly associated with the increased risk of GC (OR = 1.39). The HOXC11 and HOTAIR expression levels in the subjects with AG genotype were much higher than those with AA genotype. In the same vein, the promoter activity of G allele was more significant than that of A allele^29^. Finally, a meta-analysis study by Tao et al. showed that the HOTAIR rs4759314 polymorphism may play a role in GC susceptibility^32^. In this case, all studies were in Chinese populations and therefore could not give an overview of its status in other populations. In contrast, we did not find any significant correlation between the HOTAIR rs4759314 SNPs and GC susceptibility. This may indicate the fact that some HOTAIR risk SNP(s) may be ancestry-specific; however, this is just a hypothesis and needs to be established, by studying this SNP in other types of cancer in Ardabil and in different (ethnic) population groups suffering from GC.

Only one haplotype in the HOTAIR (GCTA) gene was associated with the risk of GC (OR = 1.31). Studies have shown that different HOTAIR variants (e.g., rs920778, rs7958904, and rs874945) correlate with different cancers, including GC, colorectal cancer, breast cancer, and esophageal cancer^33^. Knockdown of HOTAIR can prevent cell growth of GC, influence cell cycle distribution, and improve P21 and P53 protein levels^15^.

HOTTIP knockdown in GC cells hindered cell proliferation, invasion, and migration. Additionally, HOTTIP down-regulation reduced the expression of homeobox protein Hox-A13 (HOXA13) in cell lines of GC. HOXA13 affected GC cells’ HOTTIP‑induced malignant phenotypes. Both HOXA13 and HOTTIP were up-regulated in GC tissues than adjacent noncancerous tissues^25^. In the present study, none of the HOTTIP SNPs (i.e., rs3807598, rs17501292, and rs1859168) were associated with the risk of GC. In contrast, Hu et al. showed that HOTTIP rs1859168 A > C notably was associated with a decreased risk of pancreatic cancer (PC) (CC vs. AA: OR = 0.71). The C allele of HOTTIP rs1859168 could significantly reduce the relative luciferase activity in comparison to the A allele in three PC cell lines. Therefore, the functional rs1859168 A > C polymorphism could reduce the risk of PC by downregulating HOTTIP expression^34^. This discrepancy between the two studies represents the hypothesis that some HOTTIP risk SNPs may be tissue-specific. However, further studies in different cancer cell lines are required to confirm such a hypothesis. In Hepatocellular carcinoma (HCC) patients, HOTTIP rs2071265 was related with an earlier recurrence. The HOTTIP suppression in cancer cell lines of liver decreased the rates of cell invasion and increased chemosensitivity^35^. The interaction of HOTTIP rs17501292 with MALAT1 rs619586 polymorphisms had a decreased impact on the risk of HCC (OR = 0.3)^33^.

In the present study, although none of the HOTTIP SNPs increased the risk of GC, the SNP-SNP interactions of HOTAIR with HOTTIP were strongly associated with risk of GC. The SNP-SNP interaction of HOTAIR rs17720428 TG with HOTTIP rs1859168 CC increased the risk of GC (OR = 1.76). In addition, the SNP-SNP interaction of HOTAIR rs7958904 with HOTTIP rs1859168 increased the risk of GC (rs7958904 GC-rs1859168 CC, OR = 1.85; rs7958904 CC-rs1859168 CC, OR = 1.86). Interestingly, the SNP-SNP interaction of HOTAIR rs1899663 with HOTTIP rs1859168 strongly increased the risk of GC (rs1899663 GT-rs1859168 CC, OR = 4.3; rs1899663 TT-rs1859168 CC, OR = 9.37; rs1899663 TT-rs1859168 CA, OR = 6.59). To verify the findings and validate the results, further studies in diverse ethnicities and functional analysis are required.

In our research, the stratified analysis of genetic association of the HOTAIR and HOTTIP tagSNPs with clinicopathologic characteristics (such as tumor origin and intestinal-, diffuse-, or indeterminate-types of GC) revealed no significant association in all subgroups. An important problem in GC is that the most GC patients are diagnosed at the advanced stage^36^. In the present study, which was confined to Ardabil (a very high-risk area of GC in Northwestern Iran), the 0.8, 5.2, 45.5 and 48.5% of patients were diagnosed at stages I, II, III, and IV, respectively. The frequency of each HOTAIR or HOTTIP tagSNP did not show a significant difference between patients having stage I-II and stage III-IV disease. It might probably be explained by the fact that almost all the patients (94%) recruited in the study were at the advanced stage (III-IV), having poor prognosis.

The influence of lncRNAs on microRNA function and vice versa is emerging, affecting the gene expression programs. LncRNA tagSNPs can cause or destroy miRNA binding site(s) on the lncRNA. Some LncRNAs act as molecular decoys or sponges of microRNAs, with sequestrating microRNAs favoring the expression of suppressed target mRNAs. Other lncRNAs compete with miRNAs for interacting with shared target mRNAs, causing the derepression of gene expression. They can also be precursors to the production of miRNAs for silencing target mRNAs. In contrast, little is known about the influence of microRNAs on lncRNA function. They can target lncRNAs for degradation^37,38^. Here, using bioinformatic analysis, we showed that the HOTAIR rs17720428/rs7958904 and HOTTIP rs17501292/rs1859168/rs3807598 tagSNPs could lead to miRNA target gain and/or loss. However, for the HOTAIR rs1899663 and rs4759314 tagSNPs, no miRNA target gain or loss was recognized. Among the miRNAs listed in Table 9, for a small number, the functional role has been recently determined to a somewhat large extent in cancer, although not necessarily in GC, including the miR-615-3p, miR-874-5p, miR-506-5p, miR-769-3p, miR-1252-5p, and especially miR-216a-5p. For example, miR-615-3p can promote the epithelial mesenchymal transition (EMT) and metastasis of breast cancer by targeting protein interacting with C kinase 1 (PICK1)/TGFBRI axis^39^. MicroRNA-874-mediated inhibition of the major G1/S phase cyclin, cyclin E1 (CCNE1) does not occur in osteosarcomas. It also inhibits tumor metastasis in hepatocellular carcinoma by targeting the δ opioid receptor (DOR)/epidermal growth factor receptor (EGFR)/extracellular signal-regulated kinase (ERK) pathway^40,41^. MiR-506 inhibits the proliferation and invasion of i) colorectal cancer by targeting ubiquitin-like with plant homeodomain and RING finger domains 1 (UHRF1) via the KISS1/PI3K/NF-kB signaling axis and ii) nasopharyngeal carcinoma by targeting Forkhead box Q1 (FOXQ1), and is also epigenetically silenced in pancreatic cancer^42–44^. During Reoxygenation microRNA-769-3p down-regulates N-myc downstream-regulated gene 1 (NDRG1) and enhances Apoptosis^45^. By targeting miR-1252-5p, the lncRNA AL161431.1 can facilitate cellular proliferation and migration via MAPK signaling in endometrial carcinoma^46^. The function of miR-216a-5p has also been studied in depth in various cancers, playing a role of tumor suppressor. It inhibits the cell proliferation and metastasis by targeting Janus kinase 2 (JAK2)/signal transducer and activator of transcription 3 (STAT3)-mediated EMT process in GC and by targeting p21-activated protein kinase 2 (PAK2) in breast cancer. It also inhibits the cell proliferation and induces apoptosis by targeting tectonic family member 1 (TCTN1) in esophageal squamous cell carcinoma. Moreover, the low expression of miR-216a results in the upregulation of tetraspanin 1 (TSPAN1) that contributes to pancreatic cancer progression via transcriptional regulation of integrin alpha 2 (ITGA2)^47–50^.

Except for miR-615-3p and miR-1252-5p, which have lost their potential binding sites to HOTAIR (due to rs7958904 polymorphism) and HOTTIP (due to rs17501292 polymorphism), respectively and are thought to be oncogenic, the other four molecules including miR-874-5p, miR-506-5p, miR-769-3p, and miR-216a-5p play the role of tumor suppressors. MiR-615-3p and miR-1252-5p molecules may retain their oncogenic effect due to the loss of their binding site to HOTAIR and HOTTIP, respectively; however, the possible mechanism(s) is unknown and requires functional studies. MiR-769-3p has a potential binding site to HOTAIR due to the rs17720428 polymorphism associated with GC in the present study. Interestingly, all the three molecules miR-874-5p, miR-506-5p, and miR-216a-5p have a possible binding site to HOTTIP due to rs1859168 polymorphism. In the present study, the SNP-SNP interaction of HOTAIR rs1899663 with HOTTIP rs1859168 was strongly associated with GC, which may be due to the destruction of these molecules that are thought to function as tumor suppressors. However, functional studies need to be done to determine if these bindings actually occur and what the role of binding of these molecules to HOTTIP is in the progression to GC. These studies should be performed in the presence of HOTTIP rs1859168 tagSNPs by controlling the presence of HOTAIR rs1899663 polymorphism.

Taking altogether, we showed that HOTAIR rs17720428, rs7958904, and rs1899663 tagSNPs and their interactions with the HOTTIP rs1859168 polymorphism were significantly associated with GC risk. Specifically, novel SNP-SNP interactions between HOTAIR and HOTTIP tagSNPs have a larger impact than individual SNP effects on GC risk, thereby providing us with valuable information to reveal potential biological mechanisms for developing GC.

## Materials and methods

### Study subjects

A hospital-based case–control study, from October 2017 to February 2019, was conducted. A total of 300 cases were selected from patients undergoing endoscopic examination in the Imam Khomeini Hospital in the Ardabil. One control was sought for each case, frequency matched to the case group by 5-year age groups and gender. The controls were randomly selected from subjects who received routine physical examinations in the same hospital and had no self-reported history of cancer at any site. According to histopathologic and endoscopic results, gastroduodenal disease was diagnosed. GC diagnoses were categorized by anatomic sub-sites based on the International Classification of Diseases, 10^th^ Revision (ICD-10) as cardia (ICD-10 code C16.0) and non-cardia (ICD-10 codes C16.1–C16.9, involving unspecified and overlapping subsites)^51^. According to the classification of Lauren, histologic subtypes were assessed as diffuse-type, intestinal-type, and other/unspecified histologies^52^. Finally, the AJCC 8th TNM staging system for GC was considered, showing an improved efficiency in GC prognosis^53^. The study was conducted on the basis of ethical principles of human research expressed in the 1975 Declaration of Helsinki. All participants signed an informed consent form. This study was approved by the Ethics Committee of the National Institute for Medical Research Development (NIMAD)/ IR.NIMAD.REC.1396.097.

### SNP selection and genotyping

The data of genetic polymorphism from the entire sequence of lncRNAs was achieved from the dbSNP database (https://www.ncbi.nlm.nih.gov/projects/SNP/). The lncRNA HOTTIP gene sequences were downloaded by the 1000 Genomes Browser (https://www.ncbi.nlm.nih.gov/variation/tools/1000genomes/) after enlarging 2 kb of upstream and downstream flanking sequences of the gene. The selection criteria were: (i) linkage disequilibrium (LD) *r2* lower than 0.8, (ii) minor allele frequency (MAF) higher than 0.05, and (iii) the *p*-value of Hardy–Weinberg equilibrium (HWE) higher than 0.05. Seven eligible tagSNPs were chosen involving four SNPs for HOTAIR (i.e., rs17720428, rs7958904, rs1899663, and rs4759314) and three SNPs for HOTTIP (i.e., rs3807598, rs17501292, and rs17501292) eventually included in the final analysis. From each participant, venous blood samples were taken into an ethylenediaminetetraacetic acid (EDTA)-containing tube and were stored at -80 °C. Using QIAamp DNA blood mini kit (QIAGEN, Germany), genomic DNA was extracted from 200 µL peripheral blood samples as previously described^54^. All samples were genotyped by the Infinium HTS platform according to the standard protocol (https://www.illumina.com/Documents/products/workflows/workflow_infinium_ii.pdf) with a customized Illumina Infinium GSA BeadChip—a robust, high-quality assay. This SNP microarray uses known nucleotide sequences as probes to hybridize with the tested DNA sequences, allowing a qualitative and quantitative SNP analysis. Data quality control was performed using Genome Studio. The call rate cut-off was 98% as it an off-the-shelf array.

### Statistical and bioinformatic analysis

Genotyping results of SNPs were evaluated for significant departure from Hardy–Weinberg equilibrium. Using Pearson chi-square test or Fisher’s exact probability (for categorical variables), the variations in frequency distribution of genotypes and demographic characteristics were assessed. The association strength was calculated applying odds ratios (ORs) and 95% confidence intervals (CIs). All genetic models were evaluated, including dominant, recessive, co-dominant, over dominant, and log additive models of inheritance for seven SNPs. Each model provides different assumptions regarding the genetic effect. Using the SNPStats (https://www.snpstats.net/start.htm), haplotype frequencies were obtained for HOTAIR and HOTTIP according to the expectation maximization algorithm. The pairwise interactions of lncRNA SNP-SNP were calculated. Statistical analyses were done by SPSS version 19.0 (IBM, Chicago, USA). The correlations between every genetic variant and clinical features of GC were investigated. The statistical tests were two-sided; *p* < 0.05 was assumed statistically significant. The potential impact of each SNP on the establishment or destruction of the miRNA binding site was analyzed using the lncRNASNP2 database^55^.

## Data Availability

The data that support the findings of this study are available from the corresponding author upon reasonable request.
